# An empirical comparison of neural networks and machine learning algorithms for EEG gait decoding

**DOI:** 10.1038/s41598-020-60932-4

**Published:** 2020-03-09

**Authors:** Sho Nakagome, Trieu Phat Luu, Yongtian He, Akshay Sujatha Ravindran, Jose L. Contreras-Vidal

**Affiliations:** Non-Invasive Brain Machine Interface Laboratory, Electrical and Computer Engineering Department, Houston, 77004 USA

**Keywords:** Neural decoding, Brain-machine interface

## Abstract

Previous studies of Brain Computer Interfaces (BCI) based on scalp electroencephalography (EEG) have demonstrated the feasibility of decoding kinematics for lower limb movements during walking. In this computational study, we investigated offline decoding analysis with different models and conditions to assess how they influence the performance and stability of the decoder. Specifically, we conducted three computational decoding experiments that investigated decoding accuracy: (1) based on delta band time-domain features, (2) when downsampling data, (3) of different frequency band features. In each experiment, eight different decoder algorithms were compared including the current state-of-the-art. Different tap sizes (sample window sizes) were also evaluated for a real-time applicability assessment. A feature of importance analysis was conducted to ascertain which features were most relevant for decoding; moreover, the stability to perturbations was assessed to quantify the robustness of the methods. Results indicated that generally the Gated Recurrent Unit (GRU) and Quasi Recurrent Neural Network (QRNN) outperformed other methods in terms of decoding accuracy and stability. Previous state-of-the-art Unscented Kalman Filter (UKF) still outperformed other decoders when using smaller tap sizes, with fast convergence in performance, but occurred at a cost to noise vulnerability. Downsampling and the inclusion of other frequency band features yielded overall improvement in performance. The results suggest that neural network-based decoders with downsampling or a wide range of frequency band features could not only improve decoder performance but also robustness with applications for stable use of BCIs.

## Introduction

Brain Computer Interfaces (BCI) record, infer and translate different parameters associated with movement from different types of brain signals to provide volitional control to prosthetic limbs, exoskeletons, computers, and even digital avatars. The part of the BCI which deciphers the user’s motor intent from recorded brain activity is typically referred to as a neural decoder. Building high-performance neural decoders is important in four different aspects: (1) usability, (2) salient feature identification and quantification, (3) understanding of the underlying neural representations^1^, and as (4) a potential metric of neural function. First, BCI neural decoders based on scalp electroencephalography (EEG) are being designed for assistive and therapeutical applications for patients with motor disabilities in order to promote plasticity and facilitate rehabilitation^2,3^. Thus, higher accuracy in decoding performance determines the usability of the system^4^. Second, many neural features (e.g., time and frequency domain features, channel locations, channel and source domain features, to name a few^5,6^) are likely to contain varying information about motor intent and thus are candidates for decoding human movement. However, it is often difficult to identify and quantify important features given the complexities of performing lower limb experiments in people with gait disabilities limiting the amount of high-quality data. Third, decoder calibration is often focused on maximizing decoding accuracy while neglecting the explanatory power of the decoder itself. Thus, it has been difficult to advance understanding of the representation and underlying neural mechanisms of the brain and recent advances in artificial intelligence could cast insight into this respect^7^. Studies suggest that building a neural decoder using deep learning could cast insights into this aspect by deciphering its neuron layers^1,4^. This approach may enable us to study and quantify features that are relevant to the decoding task that could also help us understand the underlying mechanisms of the brain. At last, the accuracy of neural decoding could also reveal the amount of information explained by the model. It is a well-known fact that not only the cerebral cortex but also the cerebellum and spinal cord all play a crucial role in ambulatory movements. It is of interest to understand how much information we could extract from the cerebral cortex using non-invasive technology.

Restoration of gait function has been a long-standing focus of rehabilitation research and it is still an active research area to be explored^8,9^. Although there are various approaches in this domain, one of the promising approaches is to use a neural decoder to build BCI applications and understand the underlying mechanisms of the brain associated with walking^10–12^ However, the number of studies using a decoder to understand the cortical networks during gait is still very limited. Therefore, it is important to build a decoding framework that could be open-sourced and be easily deployed into such applications that could be beneficial for studying the brain of patients with disabilities.

Another application of BCIs in this context is to incorporate the interface into real-time control of assistive devices that could help people with lower limb disabilities walk again. In this case, the accuracy of the decoding performance is crucial as it determines the usability of the system. The robustness of the algorithms is also important since the likelihood of decoding errors increases when the system is used in a real-world setting.

Neural signals are nonlinear and nonstationary^13^. However, many decoding algorithms used today are based on linear models, and the features used for lower limb decoding remain at the early stage where simple filtered frequency bands are used for decoding^14–17^. Recent studies in our lab showed an implementation of non-linear real-time decoding using an unscented Kalman filter (UKF) with delta-band EEG as a feature of neural activity^12,18^. Although this improved decoding performance, it raised several questions to be explored as described below.

Previous research has shown the feasibility of using EEG to decode joint angle kinematics^16,19,20^. Presacco *et al*.^16^ demonstrated that the decoding performance of joint angles from EEG was comparable to those using multiple single-unit activities recorded in nonhuman primates. They identified the optimal number of electrodes for the decoder and observed that the fronto-posterior cortical networks were heavily involved in gait. Luu *et al*.^20^ showed the feasibility of using a closed-loop BCI to control a walking avatar under both normal and altered walking conditions while participants were introduced to visuomotor perturbations involving cortical adaptations. Luu *et al*. also demonstrated the use of a non-linear neural decoder using an unscented Kalman filter to decode joint angles during human treadmill walking using delta-band EEG as the predictor^19^. The decoder they developed was robust to ocular artifacts and allowed for real-time implementation. Similarly, Hikaru *et al*. developed a decoder to estimate muscle synergies and individual muscle activation from delta-band EEG as well, revealing the cortical correlates of muscle synergy activation associated with location^21,22^.

However, these studies have certain limitations that we intend to address in this paper. Most of the prior studies have used specific tap sizes for estimating the instantaneous joint angles without comparison. Additionally, none of the studies explored the ideal tap size for continuous decoding within the real-time implementation. Even though most of these studies claim high decoding performance based on r-value, a higher r-value does not always imply perfect tracking. Especially since a prediction which follows the general trend, but is way off from the actual values would still contain a high r-value. Also, no studies have compared the performance of using different models and/or filters for continuous decoding, and all the above cited studies made use of only delta-band power for joint angle prediction. Therefore in this study, we also explore the role that other frequency band bands may have on decoding performance.

We performed the experiments to prove the above mentioned points using online equivalent preprocessing and offline decoding combinations. It is true that online preprocessing and online decoding combinations are more idealistic, but to rigorously test a wide variety of combinations of parameters and decoders to compare against each other, we chose this approach. To make our approach feasible, we included the same preprocessing and decoder combination we have previously used in a real-time closed-loop experiment^18,19^ and treated the data in a similar manner.

The overall goal of this paper is to investigate what kind of machine learning algorithms, under what condition, perform best for EEG-based gait decoding. Although online and offline decoding is different schemes, we believe one of the advantages of offline decoding analysis is to rigorously test different conditions in order to provide feasible options in the later design of an online decoder. In this context, we investigated how the following factors affect decoding performance: (1) algorithms, including the number of hidden units, (2) tap sizes, (3) downsampling effects, and (4) frequency band features. To address the above issues, we designed and conducted offline experiments rigorously comparing performance against each other to validate the aforementioned factors.

## Methods

### Data

The data set consisted of EEG and kinematics data from 8 healthy subjects with each subject undergoing three trials that were spread across two days. Each subject walked on a treadmill for a total of 20 minutes per trial. Each trial consisted of three different tasks: resting, (based on kinematic measurement using goniometers, “Gonio”) Gonio control, and closed-loop BCI control (hereafter BCI control). Two minutes of baseline period where subjects were instructed to stand still on a treadmill was collected in each trial before and after the treadmill walking. In the beginning part of the experiment where subjects finished baseline period, subjects were instructed to walk on a treadmill at a fixed slow speed of 1 mile per hour (mph) and staring at the screen in front of them at the same time where real-time feedback of a virtual reality avatar was provided. The virtual reality avatar’s lower limb movements were synced with the goniometers attached to the hip, knee, and ankle of the participants. During the task, subjects were instructed to walk steadily for 15 mins where the decoder is calibrated for the next BCI control. Following the Gonio control, the experiment switches to a BCI control where the right leg of the virtual reality avatar is now controlled using EEG to give real-time feedback to the subject. This phase of the trial continued for five mins. A 64-channel active EEG electrode system from BrainVision was used out of which 4 channels were used as electrooculogram (EOG) sensors to capture and remove eye related artifacts using adaptive filtering algorithms^23^. The sampling frequency was set to 100 Hz. The data set was collected in our previous experiments and is publicly available with a full description^24^.

#### Train, validation, test split

The data set was split into “train”, “validation”, and “test” sets in a sequential manner to simulate the online decoding scheme. For real-time decoding, each trial consisted of two modalities: Gonio control and BCI-control^18^ (As described in the above Data section). Similarly, in our offline experiments, we utilized Gonio control to be the “train” and “validation” sets, where the first 80% was used as the “train” set and the last 20% was used as the “validation” set. The Gonio control section had 60 channels by 15 mins x 60 seconds x 100 Hz = 90,000 time samples. The BCI control phase followed the Gonio control section. This section utilized the decoder trained during the Gonio control phase and used the model to decode the right leg in real-time using the EEG signals. This section had 5 mins of data and we used this entire section as the “test” set. The BCI control had 60 channels by 5 mins x 60 seconds x 100 Hz = 30,000 time samples. In our offline experiments, the “train” data set was used to train the model with certain hyperparameters, the “validation” set was used to assess the hyperparameter combinations used in the “train” data set. Finally, the “test” data set was used to assess the best hyperparameter combinations determined by the “validation” set.

### Code

The code is available on github: https://github.com/shonaka/EEG-neural-decoding. To replicate the environment, the Anaconda virtual environment and the docker image are also available for replicating the building environment: https://hub.docker.com/r/snakagome/research_gpu.

### Metrics

To quantify the decoding performance, two metrics were used: (1) Pearson’s correlation coefficient (r-value) and (2) Coefficient of determination (R2 score). In the following equations, *y* is the actual joint angle and $\hat{y}$ is the predicted joint angle.

Pearson’s correlation coefficient (r-value) was used in our previous studies to measure performance^18,25^.

$${\rho }_{y,\hat{y}}=\frac{cov(y,\hat{y})}{{\sigma }_{y}{\sigma }_{\hat{y}}}$$

where *cov*(*X*, *Y*) is the covariance between the two variables and *σ*(*X*) is the standard deviation.

The Coefficient of determination (R2 score) is another statistical metric used to measure the degree of variation of one data series that can be predicted from another. The formulation for R2 score is not to be confused with the squared Pearson’s correlation coefficient. The value can be negative if the model overfits the training set and accounts for the variance accounted for by the model. Generally, r-value (Pearson’s correlation value) would be a useful metric if the overall trends of the prediction with respect to the ground truth are of interest. However, if you want to quantify more precise errors between the two variables (prediction vs ground truth), the R2 score is a more suitable measurement. The R2 score was also used to evaluate similar decoding tasks using invasive data in previous studies^4,26^.

$${R}^{2}\equiv 1-\frac{{\sum }_{i}{({y}_{i}-{\hat{y}}_{i})}^{2}}{{\sum }_{i}{({y}_{i}-E9)}^{2}}$$

where *E9* is the mean of the actual joint angle and *y*_*i*_ is the actual joint angle at time sample *i*.

### Pre-processing and experimental designs

**Figure 1:**
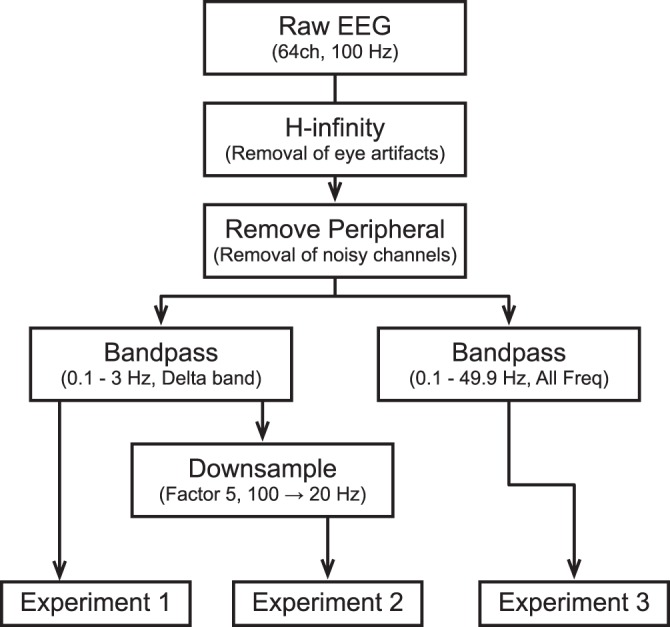
A preprocessing pipeline for two experiments. Experiment 1 in the first column is equivalent to the real-time decoding preprocessing pipeline used in the earlier study. Experiment 2 in the second column is similar to the Experiment 1, but the last preprocess bandpassed to contain all the available frequency range.

Pre-processing pipelines for different offline experiments are represented in Fig. 1. The base pipeline is selected such that they can easily be used in an online real-time decoding scheme^18^. An H-infinity algorithm was used to specifically remove eye blinks, eye motions, amplitude drifts and recording biases simultaneously^23^. The parameters of the H-infinity algorithms were kept the same as the real-time decoding. Peripheral channels were removed as they typically contain many artifactual components. The signals were then bandpass filtered using a 4th order butterworth filter. Although the frequency range was the same, this is one of the differences compared to the real-time decoding as the real-time implementation utilized finite impulse filter and the phase shift was expected. To this point, all processing was done through a MATLAB script, which is also provided in the open-sourced repository. Additionally, before each experiment, the signals were z-scored for each channel.

#### Experiment 1: Decoding based on delta band features

The protocol for Experiment 1 is equivalent to the real-time decoding pipeline used in the previous studies^18,25^. This is the baseline data processing pipeline, which will be used as a comparison for the following two experiments. We first calculated the performance metrics for each trial. We then calculated the median value for each tap size for each algorithm to draw a marker for visualization on figures. The error bars were also calculated and plotted using 25th to 75th percentile range.

#### Experiment 2: Downsampling effect

The primary goal of Experiment 2 was to investigate the effect of downsampling on delta band band-passed time samples. EEG data were resampled from 100 Hz to 20 Hz. Similar to Experiment 1, the median performance was calculated across all trials. To see the difference in performance as compared to Experiment 1, the performance of Experiment 1 was subtracted from the performance of Experiment 2. The black line for zero was added to see which Experiment performed better at certain tap sizes.

#### Experiment 3: Other frequency bands

The primary goal of the final experiment 3 was to investigate the effect of using all the frequency bands as opposed to just using the delta band features. We utilized the same bandpass filtering parameters except with a modified frequency range (0.1–49.9 Hz). As with Experiment 2, a similar analysis was performed to assess the effect of automatic feature learning from different frequency bands.

#### Tap sizes

**Figure 2:**
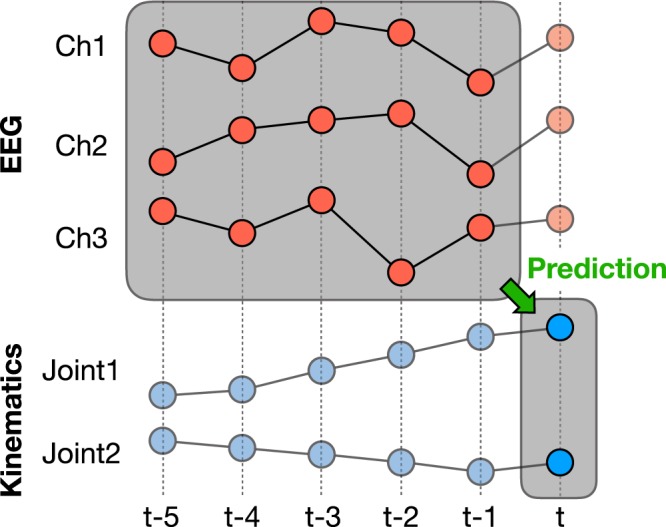
Example decoding schematic for tap size = 5. When running multiple experiments, the tap sizes ranged from 1, 2, 5, 10, 20, 30, 40, 50. Given the sampling frequency of 100 Hz, this corresponds to 10, 20, 50, 100, 200, 300, 400, 500ms, respectively.

Tap size refers to the number of samples in history used to train the model. A decoding schematic explaining the concept of decoding using a sliding window is presented in Fig. 2. The figure shows an example where the tap size is five. The tap size of five was also the tap size we used in real-time decoding to collect the data^18^. In this paper, to thoroughly test the effect of tap sizes, we tested the model with different tap sizes: 1, 2, 5, 10, 20, 30, 40, 50. Given that our sampling frequency was 100 Hz, this is equivalent to using a tap size of 10, 20, 50, 100, 200, 300, 400, 500 ms of past data to predict the 10 ms future. This was common in both Experiments 1 and 3. On the other hand, in Experiment 2, we only utilized tap sizes until 20. Considering the fact that the downsampled frequency is now 20 Hz, the tap sizes in the downsampled scenario correspond to 50, 100, 250, 500, 1000 ms for 1, 2, 5, 10, 20 tap sizes, respectively.

### Algorithms

The following eight algorithms were compared against each other: Linear regression (LR), Ridge regression (RR), Unscented Kalman Filter (UKF), CatBoost (CB), Temporal Convolutional Network (TCN), Long Short Term Memory (LSTM), Gated Recurrent Unit (GRU), and Quasi Recurrent Neural Network (QRNN). Within each architecture, the hyperparameters were listed below in each algorithm section. The hyperparameters were optimized using Bayesian optimization, which will be described in detail in the next subsection.

#### Linear Regression (LR)

Linear Regression is one of the most basic machine learning methods typically used to model the predictive relationship between the dependent target variable to multiple explanatory variables^27^. Note that in this context, Wiener Filter is equivalent to LR because of the way we feed in the input as a time sequence manner. However, in the machine learning context, we are denoting this as LR.

#### Ridge Regression (RR)

Ridge Regression is a linear regression with L2 regularization^28^. RR is equivalent to WienerRR in this context. The parameter optimized during the training was *α*, which determines the strength of the regularization. It performs regularization so that the features that influence the target-dependent variable the least get penalized the most.

#### Unscented Kalman Filter (UKF)

Unscented Kalman Filter is an improved version of the Kalman Filter^29^. It utilized an unscented transform to incorporate non-linearity within the model. In this specific context, we are following the implementation from Li *et al*. where UKF was first used to decode the kinematic movements invasively with monkeys^30^. The parameters optimized here are *λ*_*F*_, *λ*_*B*_, and *κ*.

#### CatBoost (CB)

Catboost is one of the most recent gradient boosting algorithms over decision trees^31^. The parameters optimized here are the learning rate, depth, and L2 regularization term. This was initially employed to compare against other gradient boosting algorithms, so we only picked the parameters common to these algorithms. Also, even with GPU capability, gradient boosting optimizations take a long time. There is still room for optimizing this algorithm given other parameters that we did not optimize.

Although we also implemented XGBoost^32^ and LightGBM^33^, we did not observe much of the performance difference between the gradient boosting algorithms. Since catboost was the fastest when computing using a GPU, we decided to remove both XGBoost and LightGBM from further analysis. However, the implementation is readily available on the Github link.

#### Temporal convolutional network (TCN)

Temporal Convolutional Network (TCN) is a specific type of convolutional neural network (CNN) architecture where a dilated causal convolution is wrapped with a residual block^34^. The authors compared the performance against other well known RNN architectures such as ordinally RNN, LSTM, and GRU and showed superior performance across all tasks^34^.

For all the neural network types of architectures from here below, there were common parameters that were optimized. First, the optimizer was optimized among ADAM, Stochastic Gradient Descent (SGD) with momentum, and AdaBound^35^. The learning rate and weight decay were also optimized. In addition, the number of epochs was also optimized as this is subject to change with the other parameters such as learning rate and weight decay. Specifically for TCN, the number of filters, layers, and kernel size were also optimized.

#### Long short term memory (LSTM)

Long Short Term Memory is a sophisticated version of recurrent neural networks (RNN) where three gates are added to control the information to retain and pass^36^, while avoiding the problem of vanishing gradient typically associated with training of a regular RNN.

In addition to the common parameters in the TCN, recurrent neural networks (LSTM, GRU, QRNN) had a number of hidden units, layers, standard deviation for layer initialization, and clipping strength (which helps to prevent the gradient from exploding) were optimized. We did not observe the gradient exploding in TCN so this was omitted from the TCN optimization.

#### Gated recurrent unit (GRU)

Gated Recurrent Unit is another improvement to the RNN where it has two gates to control how to retain and pass information between the nodes. The same parameters were optimized as LSTM^37^.Even though previous empirical evaluations^38^ have not shown a clear winner between GRU and LSTM, it is speculated that GRU could be a better model when dealing with a lower number of data to generalize upon, considering the fewer number of parameters in comparison to LSTM.

#### Quasi recurrent neural network (QRNN)

Quasi Recurrent Neural Networks (QRNN) is another alternative to a normal RNN where computations can be performed in parallel rather than sequential using convolutional layers^39^. The sequential dependencies in QRNN are handled using pooling, which makes the algorithm efficient to compute. The original paper that proposed the method showed its superior performance when compared against LSTM in a language modeling task. As for the actual implementation of the QRNN, we utilized QRNN implementation in fastai library^40^. The same parameters were optimized as with the LSTM.

### Hyperparameter optimizations

A Bayesian optimization library called Optuna^41^ was used in this study. A number of trials was set to the default of 100 except for RR, where only one parameter had to be tuned (trials = 50). Optuna also provides an automatic early stopping for unpromising trials to save time, which is called pruning. For pruning, an asynchronous successive halving algorithm was used with default parameters. In all the optimizations, the mean squared error was chosen as the metric to be minimized.

### Post-analysis

After testing the model with test data, the following post-analysis was performed to investigate the patterns of preference of the two best decoding algorithms and the feature of importance in all the algorithms.

#### Determination of the optimal number of layers and hidden units

To investigate the patterns of performance for the two best algorithms (GRU and QRNN), we conducted a grid search fixed number of layers and hidden units analysis for a tap size equal to five (equivalent to the real-time implementation). We optimized for the other parameters with the fixed number of layers and hidden units using optuna with 100 trials. 100 combinations were created where the number of layers differed from one to ten layers and the number of hidden units ranged from 8 to 80 with an incrementation of 8 for each step (8, 16, 24, 32, 40, 48, 56, 64, 72, 80). This way, each combination is optimized for the highest performance for the specific combination of the number of layers and hidden units. This is to make sure to exclude the possibility that certain combinations of the number of layers and hidden units favor certain hyperparameters.

After computing the 100 combinations of the number of layers and hidden units with 100 trials of hyperparameter optimizations for each combination, we plotted the average performance for each combination in a grid heatmap for each metric to study the pattern or the tendency of GRU and QRNN.

#### Feature of importance

To fairly compare the decoding models with a single feature of importance model, we utilized channel-by-channel input perturbation on a trained model. For this, we utilized the model with the tap size = 5 because (1) this was the particular tap size we used in real-time decoding, (2) to shorten the time (the analysis takes a long time even for this single tap size as it has to perform one testing per channel and there were 46 channels). We fed the data with a single channel randomly permutated in the time samples. The random seeds were fixed throughout the experiments for replicability. The performance was assessed after testing (making predictions) with the single-channel perturbation and the same process was repeated for all the channels one-by-one. By subtracting the ground truth where all the channels were not perturbed as we performed for the assessment of Experiment 1, we could evaluate the feature of importance in channels where the performance significantly drops. We also sorted the channels in order so as to pick the top five channels where the performance decreased the most, which indicates the importance of the channel.

## Results

### Experiment 1: A comparison of different algorithms

**Figure 3:**
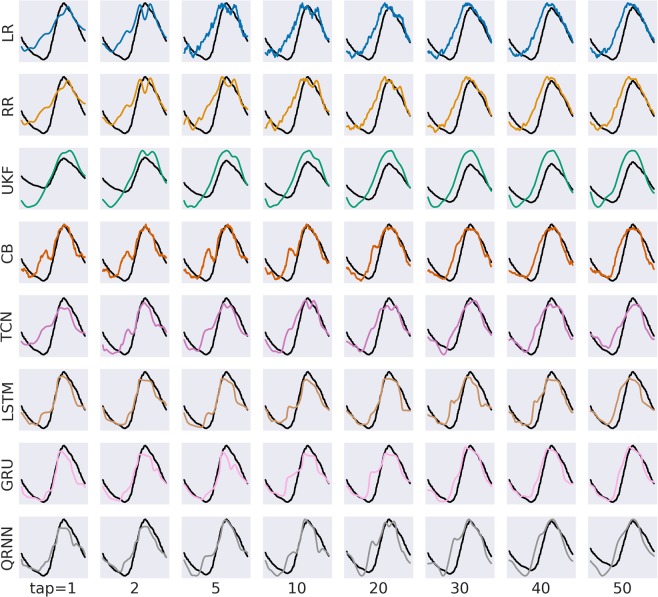
Example decoding for a single gait cycle for hip joint for each tap length resulting from Experiment 1 (delta band feature). Rows show different decoding algorithms and columns show different tap sizes used to train the model. Black lines are the ground truth from an actual joint angle measured with goniometers. For knee and ankle joints, plots are available in the Appendix.

To assess how different algorithms perform with the same pre-processing pipeline as the real-time EEG decoding, we performed a rigorous comparison based on variable tap sizes and quantified the performance based on r-values and R2 scores. Fig. 3 shows an example of the decoding results of the best subject for each algorithm with different tap sizes. Each row represents different decoding algorithms in different colors. Each column represents different tap sizes. The predicted joint angles using different algorithms tend to become more smooth and close to the actual joint angles in the black line. When focused on each algorithm, we observed that the linear decoders (LR and RR) tend to be noisy when compared to other algorithms. UKF has the smoothest curve compared to all the other algorithms but tends to be off from the ground truth. GRU, the best algorithm among the compared algorithms, aligns well with the ground truth after the tap size of 30.

#### Evaluating from r-value perspective

**Figure 4:**
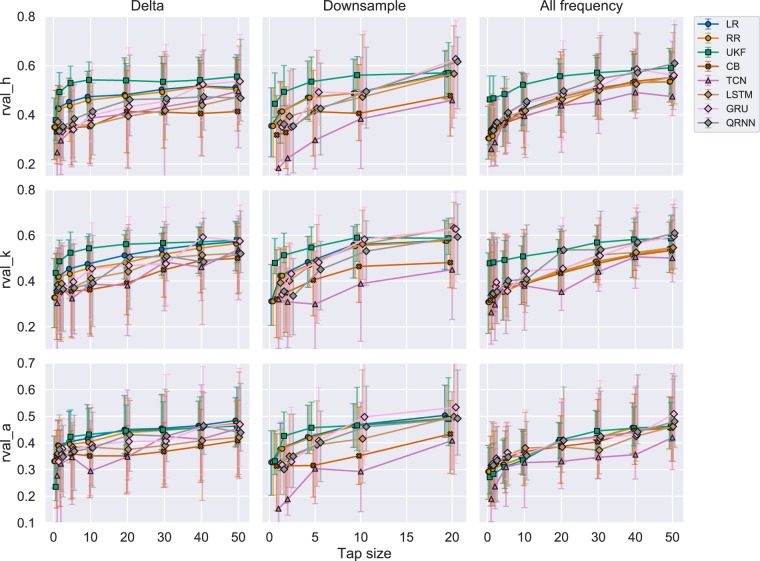
R-values for each experiment across all the joints. Each row represents each joint angle for the right leg. Each column represents each experiment. Each point represents a median performance across all the trials and subjects. Each errorbar represents 25th to 75th percentile. Different decoding algorithms are differentiated using colors. Similar algorithms were grouped using shapes. Linear algorithms with a circle (LR: Linear Regression, RR: Ridge Regression), Adaptive filter with a square (UKF: Unscented Kalman Filter), Boosting with a cross (CB: CatBoost), CNN with a triangle (TCN: Temporal Convolutional Network), RNN with a diamond (LSTM: Long Short Term Memory, GRU: Gated Recurrent Unit, QRNN: Quasi-Recurrent Neural Network).

 Figure 4 shows a comparison of performances among each algorithm measured by r-value. Each row represents different joint angles and each column represents different experiments as described in Fig. 1. In this section, we are specifically focusing on the first column. Each marker represents a median r-value across all the subjects and trials. The marker shapes represent similar algorithms with a circle denoting linear algorithms, square for Kalman filters, cross for boosting, triangle for CNN, and diamond for RNNs. All the algorithms tend to increase their performance as the number of tap sizes increased. This is apparent in the errorbar range of 25th to 75th percentile as the range also increased the performance. UKF showed superior performance across different tap lengths for the hip and knee joint reaching an average r-value of more than 0.50. LR and RR showed superior performance across different tap lengths for the ankle joint reaching an average r-value of more than 0.45. On the other hand, CB, TCN, and LSTM performed worse in this metric.

UKF also reached 90% of the accuracy in r-value with respect to the maximum r-value across all the tested tap sizes when it reached the tap size equal to five. LR and QRNN also reached their 90% of the maximum accuracy after a tap size of 20. Other algorithms tend to require larger tap sizes as the accuracies continued to grow even after a tap size of 50.

#### Evaluating from R2 score perspective

**Figure 5:**
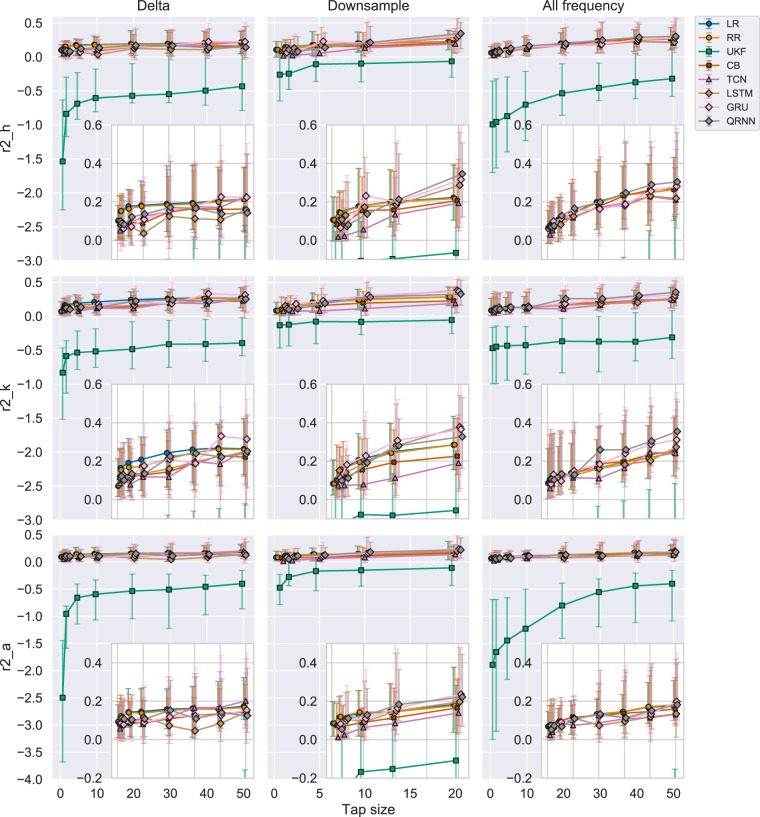
R2 scores for each experiment across all the joints including UKF. Each row represents each joint angle for the right leg and each column represents each experiment. Each point is a median of R2 score across trials and subjects. Each errorbar represents 25th to 75th percentile. The inset figures are the magnified version to compare algorithms other than the UKF. Different decoding algorithms are differentiated using colors. Similar algorithms were grouped using shapes. Linear algorithms with a circle (LR: Linear Regression, RR: Ridge Regression), Adaptive filter with a square (UKF: Unscented Kalman Filter), Boosting with a cross (CB: CatBoost), CNN with a triangle (TCN: Temporal Convolutional Network), RNN with a diamond (LSTM: Long Short Term Memory, GRU: Gated Recurrent Unit, QRNN: Quasi-Recurrent Neural Network).

 Figure 5 shows a comparison of performances among each algorithm measured by R2 score with and without UKF, respectively. We are specifically focused on the first column for Experiment 1 where each marker represents a median R2 score across all the subjects and trials. Each row represents different joint angles. UKF significantly underperformed as compared to other algorithms (Fig. 5). We observed that the LR and RR outperform other algorithms with smaller tap sizes, but this was overcome by other algorithms such as GRU and TCN as tap size increased.

### Experiment 2: downsampling effect on decoding performance

Experiment 2 from Fig. 1 was performed to investigate the effect of downsampling on performance. In the following sections, we assessed the performance using the two metrics and comparing against the baseline data processing pipeline in Experiment 1.

#### Evaluating from r-value perspective

Figure 4 in the second column shows the r-value performance for the Experiment 2. We still see the UKF dominates the performance in the smaller tap sizes up to 10 in hip and knee and 5 in the ankle, but GRU and QRNN start to perform better as tap size increased. The performance of CB and TCN in this experiment still comparatively under performed, as in experiment 1.

**Figure 6:**
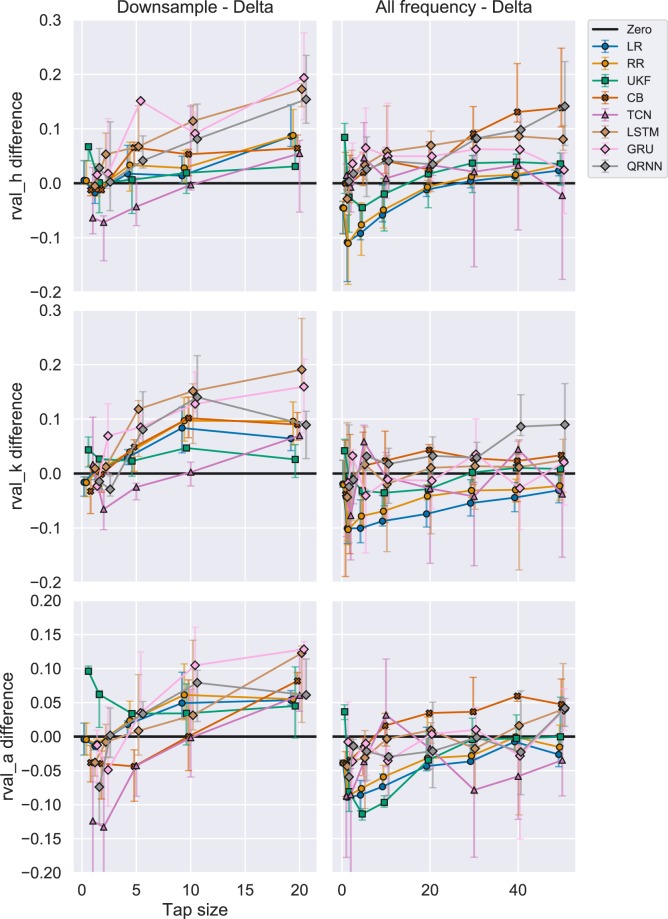
R-values difference between (1) Downsample vs Delta and (2) All frequency vs Delta. Each row represents different joint angles for the right leg and each column represents the differences between (1) Experiment 2 vs (1, 2) Experiment 3 vs 1 in performance (r-value). The black line shows a zero threshold to indicate which experiment performed better at a certain tap size. Similar algorithms were grouped using shapes. The errorbar shows 25th to 75th percentile and the middle marker shows the median value. Linear algorithms with a circle (LR: Linear Regression, RR: Ridge Regression), Adaptive filter with a square (UKF: Unscented Kalman Filter), Boosting with a cross (CB: CatBoost), CNN with a triangle (TCN: Temporal Convolutional Network), RNN with a diamond (LSTM: Long Short Term Memory, GRU: Gated Recurrent Unit, QRNN: Quasi-Recurrent Neural Network).

To clearly see the difference in performance compared to Experiment 1, we subtracted the performance of Experiment 1 from the performance of Experiment 2, which is displayed in Fig. 6 in the first column. The black line represents zero performance difference between the two experiments. We could see the performance in most of the algorithms increased with the downsampling except for the TCN in the smaller tap sizes. In fact, RNNs (LSTM, GRU, and QRNN) improved its performance more than 0.1 in r-value for hip and knee, and 0.05 in r-value for ankle joint. UKF had a unique trend where the performance increase was at its highest for tap size = 1 and slowly reduces to a plateau of performance at 0.025 as the tap sizes increased.

#### Evaluating from R2 score perspective

Figure 5 in the second column shows the R2 score for Experiment 2 with and without UKF, respectively. As represented in Fig. 5, UKF performed the worst compared to all the other algorithms. To clearly show the other algorithms performance, we also included inset figures that magnify the other algorithms for comparison. In this experiment (2nd column), we could observe the RNNs (LSTM, GRU, QRNN) performed well compared to other algorithms across different tap sizes except for the smallest of tap sizes (1, 2, and 5) and certain joints (hip and ankle).

**Figure 7:**
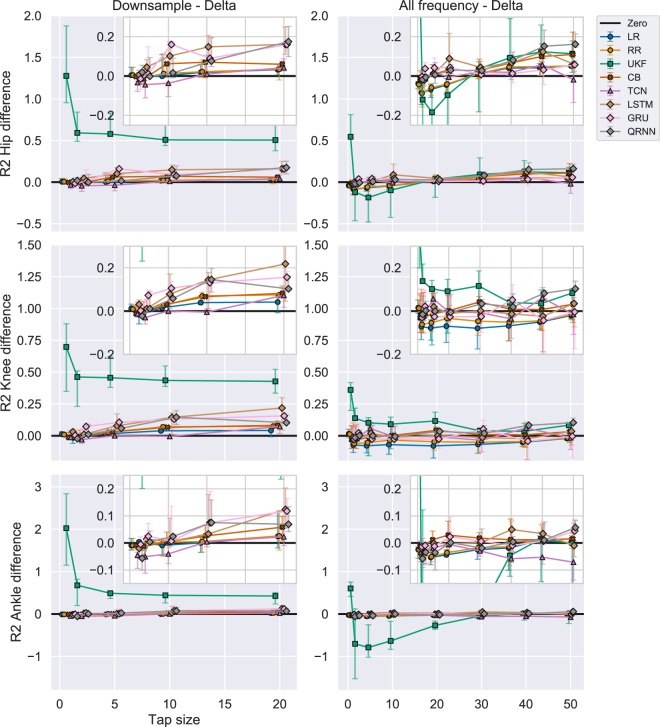
R2 difference with UKF between (1) Downsample vs Delta and (2) All frequency vs Delta. Each row represents different joint angles for the right leg and each column represents the differences between (1) Experiment 2 vs (1, 2) Experiment 3 vs 1 in performance (R2 score). The black line shows a zero threshold to indicate which experiment performed better at a certain tap sizes. Similar algorithms were grouped using shapes. The errorbar shows 25th to 75th percentile and the middle marker shows the median value. The inset figures show a magnified view to compare different algorithms other than the UKF. Linear algorithms with a circle (LR: Linear Regression, RR: Ridge Regression), Adaptive filter with a square (UKF: Unscented Kalman Filter), Boosting with a cross (CB: CatBoost), CNN with a triangle (TCN: Temporal Convolutional Network), RNN with a diamond (LSTM: Long Short Term Memory, GRU: Gated Recurrent Unit, QRNN: Quasi-Recurrent Neural Network).

To evaluate the performance difference in R2 score compared to Experiment 1, we compared the R2 score by subtracting the performance of Experiment 1 from the performance of Experiment 2 (Fig. 7 in the second column). The black line represents the point of zero performance difference between the two experiments. UKF significantly increased its performance in Experiment 2 compared to Experiment 1 (Fig. 7) although the improved performance evaluated in R2 score was still the worst as compared to other algorithms (Fig. 5). The performance increase for UKF plateaued after the tap size of 2. Overall, RNNs (LSTM, GRU, and QRNN) significantly increased its performance in Experiment 2 compared to Experiment 1. The performance of CB also increased essentially consistent with an increase in tap size. Linear algorithms such as LR and RR showed a minimal increase in performance with downsampling.

### Experiment 3: automatic feature learning in neural networks

Experiment 3 (see Fig. 1) investigated how frequency band features, other than the delta band, affect decoder performance. Similar to Experiment 2, we assessed the performance based on the two metrics.

#### Evaluating from r-value perspective

In the third column of Fig. 4, we see the performance changes with different tap sizes for Experiment 3. The trend was very similar to Experiment 2, where UKF initially performed well for hip and knee joints where the tap sizes were small, but then GRU and QRNN outperforms the UKF. One difference compared to Experiment 2 is that, for the ankle joint, GRU and QRNN always performed better than the UKF in all the tap sizes.

To evaluate the performance difference compared to Experiment 1, we subtracted the performance of Experiment 1 from Experiment 3 and showed the difference in Fig. 6 in the second column. One interesting trend in the performance difference is that the linear decoders (LR and RR) and UKF tend to perform worse when all the frequency was used. This effect is minimized as the tap size increased. On the other hand, boosting algorithms (CB) and neural networks gained a slight increase in performance (around 0.05 in r-value).

#### Evaluating from R2 score perspective

Figure 5 in the third column shows the performance evaluated for the 3rd experiment using R2 score. Again, UKF did not perform well when evaluated from R2 score. In the third column of the same Figure, QRNN performed well across different tap sizes for hip and knee joints. Ankle joints showed similar performance among different algorithms where it was difficult to conclude which algorithms performed the best.

To evaluate the performance difference compared to the Experiment 1, we subtracted the R2 score of Experiment 1 from Experiment 3 and showed the difference in Fig. 7 in the third column. In Fig. 7, UKF showed a significant increase in performance in tap size = 1, but the Experiment 3 with all the frequency band features performed well in tap sizes 2, 5, 10 for hip and 2, 5, 10, 20, 30, for ankle joint. The only exception was the knee joint where the performance in Experiment 3 for UKF was still superior compared to the R2 score in Experiment 1.

Figure 7showed an interesting pattern where the performances of linear algorithms such as LR and RR did not increase in Experiment 3 as compared to Experiment 1 in smaller tap sizes (1, 2, 5) for hip, all the tap sizes except 1 in the knee, and tap sizes until 30 in the ankle joint. CB constantly showed a performance increase across different tap sizes and joints. The performance of TCN did not increase for the hip and knee joints and decreased for the ankle joint. RNNs (LSTM, GRU, QRNN) showed the largest performance increase in the hip joint, but only some of the RNN algorithms had a performance increase in larger tap sizes for knee and ankle joint angles.

### Different number of layers and hidden units

**Figure 8:**
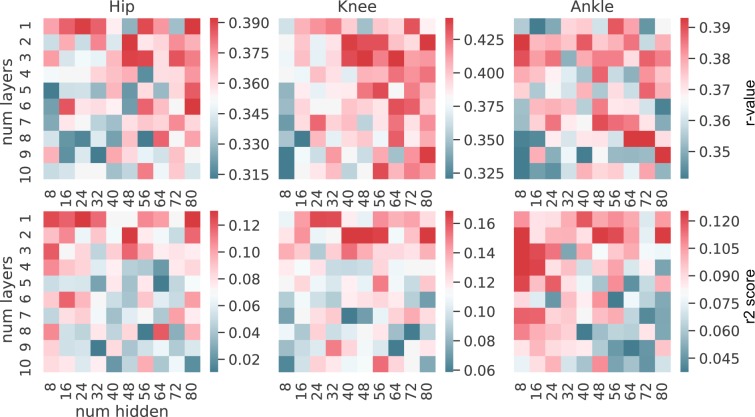
GRU assessing the number of layers and hidden units patterns. The first row represents the median r-value for each joint. The second row represents the median R2 score for each joint. The columns show each joint. The color bar indicates the median performance across trials and subjects for the specific combinations. The x-axis in each figure shows the number of hidden units and the y-axis shows the number of layers used for the model.

**Figure 9:**
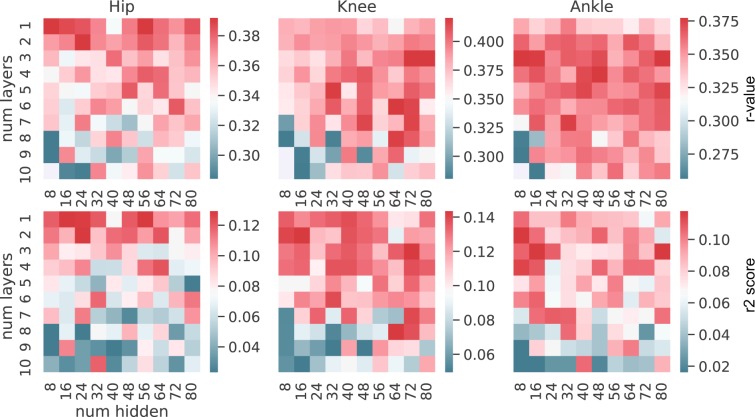
QRNN assessing the number of layers and hidden units patterns. The first row represents the median r-value for each joint. The second row represents the median R2 score for each joint. The columns show each joint. The color bar indicates the median performance across trials and subjects for the specific combinations. The x-axis in each figure shows the number of hidden units and the y-axis shows the number of layers used for the model.

For the two well-performing neural networks (GRU and QRNN), we conducted rigorous experiments to investigate the patterns of the number of layers and hidden units. For each combination of layers and hidden units, we optimized for the hyperparameters to obtain the best performance for the combination. This way, other parameters would not bias or favor some particular combination of layers and hidden units. We calculated the median performance across trials for the combination and represented them as a heatmap for each metric and each joint (Figs. 8 and 9). GRU (Fig. 8) and QRNN (Fig. 9) showed similar patterns. Overall, the performance was superior with a lower number of layers (1-5) compared to a higher number of layers (6-10). Patterns based on the number of hidden units differed between the metrics and joints. When the performance was evaluated from R2 score, general patterns suggested that a fewer number of layers and hidden units (8-40) showed better performance when compared to a higher number of layers and hidden units.

### Feature of Importance

**Figure 10:**
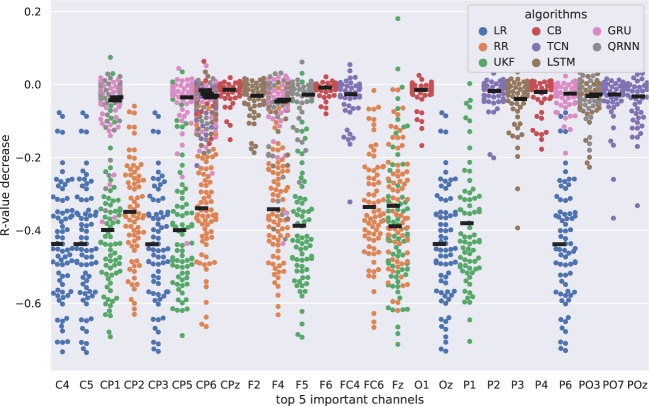
Feature of importance in channel assessment for each decoding algorithm evaluated with r-value. The x-axis shows the top five important channels for each algorithm and the y-axis shows the decrease in r-value when the specific channel was perturbed. The thick black line represents the median decrease in r-value for each algorithm for each channel.

**Figure 11:**
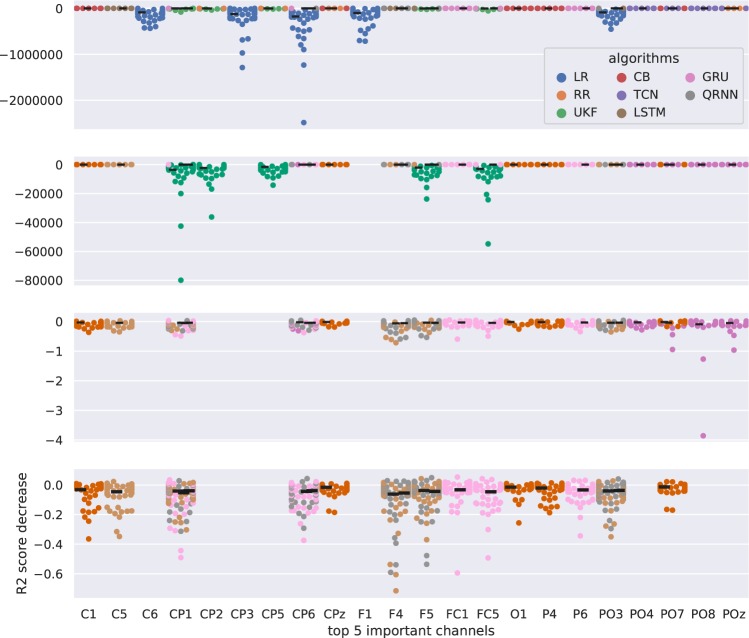
Feature of importance in channel assessment for each decoding algorithm evaluated with R2 score. There are four plots that share the same x-axis with the channel of importance after input perturbation analysis. The y-axis shows the decrease in the R2 score after the perturbation of the channel. The first plot includes all the decoding algorithms. The second plot shows algorithms without LR and RR. The third plot shows the algorithms without LR, RR, and UKF. The fourth plot shows algorithms without LR, RR, UKF, and TCN. The thick black line represents the median decrease in R2 score for each algorithm for each channel.

Feature of importance in channel analysis revealed not only the importance of some EEG channels, but the robustness of the model. Figs. 10 and 11 show the results of the feature of importance evaluated in the two metrics. Figure 10 presents the importance of channels evaluated based on the decrease in r-value with respect to the ground truth where no perturbation was performed. The x-axis shows the identified top five important channels from each algorithm and the y-axis shows the decrease in r-value when the channel was perturbed. Similarly, Fig. 11 evaluated the feature of importance using the R2 score decrease. There are four plots in this case because some algorithms significantly decreased its performance compared to other algorithms so that it was difficult to assess all the algorithms together in a single plot. All of the algorithms are represented in the 1st plot (1st row), the second plot excludes the linear models, such as LR and RR (2nd row), the third plot excludes the linear models and UKF (3rd row), and the final plot excludes linear models, Kalman filters, and TCN (4th row).

## Discussion

This study aimed to determine better algorithms for EEG gait decoding from four different aspects: (1) Algorithms, including the number of layers and hidden units, (2) Tap sizes, (3) Downsampling, and (4) Frequency band features. We computed the decoding accuracy and also assessed the EEG channel of importance and robustness of the decoder. The results identified that the former state-of-the-art algorithms (UKF) can still be one of the best algorithms when evaluated from the r-value perspective but performed the worst when evaluated from the R2 score perspective and were also vulnerable to the perturbations compared to the neural networks based algorithms. RNN based algorithms such as GRU and QRNN performed well when assessed from multiple perspectives. These algorithms were also the most robust algorithms as the perturbations in channels did not deteriorate the performance much compared to the other compared algorithms. Linear algorithms such as LR and RR are still widely used algorithms and they have their advantages as performing as the baseline. However, the performance of such algorithms significantly decreases when some channels were perturbed and this shows their vulnerability as a model.

The current study aimed to improve the decoding accuracy and robustness for the lower limb decoding using EEG from algorithm perspectives. Accurate lower limb decoding is important for controlling exoskeletons and neuroprosthetics for better usability and systems. In the previous studies, we showed the average accuracy across subjects could not exceed the r-value of 0.5^18^. With such low to mid accuracy, participants may not be able to engage in the virtual reality feedback and the neural decoding could not extend to more practical applications of lower limb movements such as controlling exoskeletons or neuroprosthetic legs where a high decoding accuracy is necessary for safety and better usability.

It is important to note that there are four different combinations of preprocessing and decoders to prove decoding performances. (1) Offline preprocessing and offline decoding, (2) Offline preprocessing and online decoding, (3) Online preprocessing and offline decoding, (4) Online preprocessing and online decoding. The current paper focuses on the third combination although the fourth combination is the most idealistic scenario. However, the fourth combination is both challenging and time-consuming because the real-time validation has to be validated in a real-time manner and thus not suitable for testing every combination of parameters and decoders. To make our study on the third combination feasible, we utilized the same preprocessing pipeline and the decoder that we have previously used in a closed-loop experiment^18,19^ and compare the performance against it to validate each method we used. We also made sure not to use any future data and using the exact same data acquired during the closed-loop experiment for training and testing, simulating the real-time applicability in future studies.

Linear algorithms such as LR and RR showed a standard performance that is not necessarily superior to other decoder algorithms but can be used as a baseline decoder prior to closed-loop BCI studies. UKF was previously used both in invasive^30^ and noninvasive^25^ real-time applications to show its capability in neural decoding. Therefore, this can be considered as the state-of-the-art in a sample by sample decoding. In this study, UKF still showed its superior performance in early convergence with smaller tap sizes and when evaluated from the r-value perspective. On the other hand, UKF showed its vulnerability when evaluated from the R2 score and also when a channel is perturbed. Although CB did not show superior performance among the algorithms compared in this study, the uniqueness of CB is that the choice of a feature of importance was different from other algorithms. Therefore, CB itself could not be the main decoder, but when ensembling the models to aim for higher accuracy, this algorithm could be considered as one of the algorithms along with better performing algorithms. Despite the previous study^34^ showing TCN could outperform RNNs, this was not the case in this study. One of the reasons may be the fact that the number of training samples fed into the models at a time was low considering that the final goal is to build a real-time application where the number of tap sizes is limited. This can be indicated in Fig.4 where Experiment 2 (Downsampling) experiment showed TCN performing the worst. On the other hand, we could observe that the R2 score of TCN becomes the best among all the other algorithms in tap size with 50 for hip and ankle joint in Experiment 1 (Delta, Fig. 5). However, from Fig. 6, when we used all the frequency bands, TCN did not perform as well, even for a tap size of 50. This could be explained by the influence of the filter size. If the filters are small, they might tend to learn local features; in our case, this would be high-frequency components. By including all frequencies, TCN will have more variance that it can explain in the high-frequency range and the majority of the filters might focus on these high-frequency components. Since deep learning models might tend to overfit and explain the maximal variance, it might weigh the higher frequencies more. This might lead to the model not focusing on the delta/ slower oscillatory components which would also have discriminatory information. On the other hand, when you force it to look into the lower frequency (using delta band alone in experiment (1), since there is no information in the higher frequencies, it will naturally look for features in the delta band and increasing the tap size would essentially increase the information present in these lower frequencies.

In the original paper, the dataset used to bench test involves data that are either highly correlated in space or contain high-frequency information. Therefore TCN should learn to capture this information with their smaller filter size and that could be the reason for having comparable results with recurrent networks. Since EEG has a lot of low frequency information, TCN might not put emphasis on this information when using lower filter size. Therefore more analysis needs to be done on filter width and how it would affect the features learned.

RNNs, especially GRU and QRNN showed superior performance from multiple perspectives. The most notable performance increase can be observed in Experiments 2 and 3 which we will discuss more details below. These algorithms also showed the robustness to channel perturbation (Fig. 11).

Generally, all the algorithms increased their performance as the tap sizes increased (Figs. 4 and 5) as expected. UKF quickly reached a plateau in performance reaching 90% of the maximum accuracy with the tap size equals five. Linear decoders such as LR and RR reaches 90% of its maximum accuracy with the tap sizes 20–30. Other neural networks kept increasing its performance as far as 50 in Experiment 1 and 3 or 20 in Experiment 2. Therefore, linear decoders and UKF tend to perform better at a lower tap size but with more tap sizes, other algorithms could outperform the linear and UKF decoders. This could be used as one of the baselines when determining the tap size to be used in real-time decoding.

Downsampling of delta bandpassed features (Experiment 2) generally increased the performance compared to the equivalent tap size performance in Experiment 1 (Figs. 6 and 7). Only exceptions were some of the small tap sizes (1 and 2) and algorithms such as TCN. From the r-value perspective, RNNs benefited from the downsampling increasing the r-values up to 0.2 for hip and knee, 0.1 for the ankle in the best case (Fig. 6). From the R2 score perspective, UKF benefited the most increasing the R2 scores significantly in the smaller tap sizes and gradually plateauing its performance increase as the tap sizes reach 20. However, even with such increases, the R2 score was still the worst compared to other algorithms (Fig. 5).

Previously, how sampling frequency would affect the performance was unknown^42^. This study investigated this issue in a sample-by-sample decoding scheme and showed that the performance could increase. We could still record the data using the highest sampling frequency, but if we were to use delta bandpassed features, we could technically reduce the sample size to 20 Hz provided that we give enough frequency range for a reconstruction. With this approach, we could technically also increase the future prediction time from 1 ms (when 100 Hz) to 5 ms (in 20 Hz) with the same decoding scheme. We also showed that the performance could actually improve.

Linear decoders and UKF decreased their performance in some cases when comparing the performance of the delta band features (Experiment 1) and all the frequency band features (Experiment 3) as represented in Figs. 6 and 7. We observed that the performance for the hip joint decreased until the tap size of 20, whereas the knee and ankle joint constantly showed a decrease in performance in both metrics (r-value: Fig. 6, R2 score: Fig. 7). UKF also showed performance decrease until tap size equals to 15 for hip, 30 for knee, and all along when evaluated from r-value (Fig. 6). Similarly, when evaluated from the R2 score (Fig. 7, the tap size equals 20 for hip and 30 for the ankle (except tap size equals to one in both metrics). On the other hand, this was not the case for other algorithms (except for TCN) where the performance increased up to 0.15 in r-values (Fig. 6) and R2 score (Fig. 7).

These results not only validate the importance of feature extraction using delta band for the performance for linear decoders and UKF, but it also shows us the boosting algorithm (CB) and RNNs could benefit from other frequency band features because of their ability to extract meaningful features for the performance.

GRU and QRNN showed similar preferences in the number of stacked layers and hidden units. We observed certain patterns of combinations of the number of stacked layers and hidden units that gave better performance compared to other combinations. For example, GRU showed better performance with less number of stacked layers when evaluated from R2 score perspective (Fig. 8). This is in line with the latest review^42^ where shallow networks were observed with intra-subject studies.

Linear decoders (LR and RR) and UKF showed vulnerability to the input perturbations when evaluated from both r-value (Fig. 10) and R2 score (Fig. 11). On the other hand, neural networks and boosting algorithms did not deteriorate their performance as much as well as the boosting algorithm (CB). This can be also considered that the RNNs and CB are more robust and not relying heavily on certain channels. CB also showed an interesting pattern where the identified feature of importance channels was unique from others. This could be beneficial when creating an ensemble model with other better performing algorithms so that the end model could not only be more robust but also perform well.

There are some channels that were identified as important from multiple algorithms such as F4, CP1 and CP6 (Figs. 10 and 11). Although it is difficult to conclude from sensor level analysis compared to the source level analysis, these channels are located near the primary motor area at the center (CP1) and posterior parietal areas (CP6) that are known to be involved during the gait. Since the subject receives feedback based on the movement of the avatar, there might still be instances of adapting to this new paradigm of walking. This might be the reason why F4 was selected as an important feature by multiple algorithms as they are reported associated with coordinating motor movements and in adapting to the gait.

The real-time applicability of the model is an important part of the design of the neural decoder. Linear models and Kalman filters such as UKF were previously shown to be implementable by many studies. On the other hand, neural networks and boosting algorithms in neural decoding context had not shown its feasibility except for a few studies in the invasive decoding^43,44^. More specifically, Sussillo et. al. used a variant of RNN called Multiplicative RNN with big data collected throughout invasive neural decoding of kinematics^43^. The performance was compared against the state-of-the-art kalman filter during the time and the RNN not only showed a superior performance but also robustness to noise which is in line with our results shown in this paper. Tseng et. al. compared the performance of a wiener filer, kalman filter, UKF, and LSTM^44^. They also reported similar trends in performance increase and robustness. The current results in our paper also prove these points and for the first time in a non-invasive context. Alternatively, the use of cloud computing is also promising as represented in Amazon Web Services (AWS) and the use of deep learning in real-time^45^.

The preprocess pipeline used in our methods is focused on real-time applicability as mentioned before in this section. Therefore, we did not use some methods such as independent component analysis (ICA) for artifact removal. In addition, artifacts are carefully taken care of in our data processing pipeline where eye-related artifacts are removed using H-infinity filter^23^, motion-related artifacts are minimized by ensuring the use of elastic mesh on the electrodes during the data collection, slow walking speed (1 mph), and the removal of peripheral channels^18,25^ Muscle related artifacts were also dealt with in a similar manner^18,25^. Although we do not claim the EEG data are completely free from the artifacts, we performed reasonable data collection and processing to minimize the effects.

As a summary and recommendation, if the purpose of the lower limb decoding requires precise control (e.g. controlling an exoskeleton), based on the high R2 score, GRU or QRNN would be recommended with shallow layers and a small number of hidden units to start with. The tap sizes could be chosen from 10 to 20 to start as that is where the performance started to plateau. Further research is required to validate the applicability of such neural networks in real-time when the training is involved. If the purpose of the decoding is to show weak trends following the ground truth, such as virtual reality feedback, UKF might be sufficient as it could provide high r-value against the lower limb movement and it has already been shown as a real-time application. In either case, simple linear models such as LR and RR could also be implemented and be used a baseline benchmark results to further improve the performance of other algorithms when tuning hyperparameters.
